# Fingerfoods: a feasibility study to enhance fruit and vegetable consumption in Dutch patients with dementia in a nursing home

**DOI:** 10.1186/s12877-020-01792-5

**Published:** 2020-10-23

**Authors:** Annemijn Visscher, Marieke C. E. Battjes-Fries, Ondine van de Rest, Olga N. Patijn, Mascha van der Lee, Nienke Wijma-Idsinga, Gerda K. Pot, Peter Voshol

**Affiliations:** 1 Department of Nutrition and Health, Louis Bolk Instituut, Bunnik, The Netherlands; 2 Division of Human Nutrition and Health, Wageningen University, Wageningen, The Netherlands; 3 ZuidOostZorg, Drachten, The Netherlands; 4 Department of Nutritional Sciences, King’s College London, London, UK

**Keywords:** Fingerfoods, Fruit and vegetable consumption, Dementia, Elderly, Nursing homes, Nutritional status

## Abstract

**Background:**

Eating problems are highly prevalent in older patients with dementia and as a consequence, these patients are at greater risk of becoming malnourished. Fingerfoods, snacks that can be picked with thumb and forefinger, could be used to counteract malnutrition in patients with dementia. The aim of this feasibility study was to evaluate whether providing fruit and vegetable rich fingerfoods in the form of recognizable and familiar snacks on top of the normal intake was feasible for both patients with dementia and caregivers as a means to increase patients’ nutritional status.

**Methods:**

Institutionalised patients with dementia (*N* = 15, 93% female, mean age = 85 years) were included in this feasibility study in the Netherlands. The residents received their regular diet supplemented with fingerfoods, comprising quiches and cakes rich in fruit or vegetables, for 6 weeks. Daily fingerfood consumption together with compensation behaviour at dinner of residents was administered with a checklist and food diaries at the start and end of the intervention as dose delivered. Furthermore, caregivers were asked to fill out a feedback form at the end of the intervention to measure fidelity and appreciation of the intervention.

**Results:**

Patients consumed on average 1.4 pieces (70 g) of fingerfoods daily, containing 41 g of fruit and/or vegetables. Fruit and vegetable consumption increased during the provision of the fingerfoods and the residents seemed not to compensate this intake during the rest of the day. The intervention was generally positively received by the majority of caregivers, depending on the type of fingerfood and state of the resident.

**Conclusion:**

This feasibility study showed that providing recognizable fruit and vegetable rich fingerfoods to patients with dementia seems feasible for both patients and caregivers and could provide a pragmatic approach to enhance fruit and vegetable consumption and total food intake in institutionalized elderly. In an up-scaled study, effects of fingerfoods on nutritional status and quality of life should be investigated.

## Background

Dementia is a neurodegenerative syndrome, characterised by impairment of memory, thinking, language, ability to perform everyday activities, and changes in behaviour and character [1–3]. In the Netherlands, at least 270,000 people have dementia, of whom 70,000 patients live in care- or nursing homes. Dementia is often associated with eating problems [4]. According to a study of Volicer et al., eating problems were found in 76% of all institutionalised patients with dementia [5]. As a result of eating problems, patients with dementia can for example seek out for foods between meals, prefer sweet foods more than before, or eat with their hands [6]. Other examples include patients who forget how to use cutlery appropriately and that patients have problems with indicating how close food should be put to their mouth [7]. In addition, patients with dementia may show defensive behaviour, which can result in refusal to eat [8].

These eating problems may lead to a low total food intake. A couple of cross-sectional studies have shown that malnutrition and risk of becoming malnourished was prevalent in 56% of elderly with dementia living in care and nursing homes, which was higher compared to elderly without dementia (46%) [9, 10]. Meijers et al. showed in a study in Dutch care homes that the prevalence of malnutrition was 22% in residents with dementia, compared to 14% in residents without dementia [11]. As a consequence of malnutrition, patients are at greater risk to have an impaired immunity, delayed healing, and micronutrient deficiencies. Other consequences of malnutrition are fractures, osteoporosis, depression, hypothermia, bedsores, and a faster loss of independence [8, 12]. These factors affect physical health of patients with dementia and may impair quality of life [4].

A low total food intake may also cause a low fruit and vegetable consumption. Fruit and vegetables contain important vitamins and minerals and a low fruit and vegetable consumption may impair quality of life, for example by causing constipation [13, 14]. In the Netherlands, 70% of the community-dwelling elderly (70 years and older) does not meet the nutritional guideline for fruit consumption and 50% does not meet the guideline for vegetable consumption. It is assumed that this percentage is even higher in elderly living in care and nursing homes, though this has not been measured [15, 16].

To counteract these health problems, caregivers need to devote a larger part of their available time to ensure adequate nutrition in patients with dementia. However, this is contradicted by the high work load and increased stress amongst caregivers working in nursing homes [17]. Nutrition for patients with dementia can even lead to increased stress for their caregivers, due to the possible defensive behaviour of patients with dementia [7, 8, 18]. In the literature, a range of nutritional interventions has been described to counteract these problems, which showed positive effects on communication and behaviour of residents during dinner, but most interventions only showed small effects on increasing food intake [4, 19, 20].

A promising nutritional strategy for patients with dementia might be the use of fingerfoods [7, 12, 20, 21]. Fingerfood is a general term for food that can be picked with thumb and forefinger; the use of cutlery is therefore not needed [12, 22]. Since patients with dementia are often unable to recognize or use cutlery correctly and therefore eating by hand, fingerfoods may lead to an increased sense of independence as they require less assistance during mealtime [12]. Additionally, fingerfoods are useful for patients with dementia who are restless, since it is not required to eat fingerfoods while sitting at the table [22, 23]. Therefore, fingerfoods can have a positive effect on quality of life, feeling of dignity, eating independence, and nutritional status of patients with dementia [12, 20]. Fingerfoods can be used to improve consumption of fruits and vegetables in patients with dementia as well [13, 24]. International studies have shown positive effects of fingerfoods on the enjoyment of eating, the amount eaten, and independence of patients with dementia [12, 20, 24–26]. However, in these studies fingerfoods were used as replacement for the main meals. To our knowledge no studies are conducted yet on providing fingerfoods as snacks on top of the main meals with the aim to increase patient’s daily food intake and specifically increase their fruit and vegetable intake.

In the current feasibility study, a small-scale fingerfoods intervention was conducted and evaluated. In this study fingerfoods rich in fruit and vegetables were provided as snack on top of the regular meals and snacks to patients with dementia living in a nursing home. We evaluated whether providing fingerfoods as an extra snack moment was feasible for both the patients and their caregivers.

## Methods

### Study design

A 6 week feasibility study was conducted between October and early December 2017 in a nursing home for elderly with dementia in the North of the Netherlands. Two different group homes within the nursing home participated in this study with a pre-test post-test design, in which dose received, fidelity and appreciation were measured as indicators of feasibility [27, 28]. This study was exempted from METC approval by the METC-WU. Written informed consent was signed by family as legal guardians of participants in this study, since the mental state of the participants (elderly with advanced dementia) made them unable to provide appropriate informed consent.

### Study population

Two group homes of one nursing home participated in this study. Patients living in these group homes were elderly with advanced dementia. Exclusion criteria for participation to the study were having a special diet or receiving enteral tube feeding which does not allow to eat fingerfoods, unable to eat solid food due to chewing and/or swallowing difficulties, and severe illness which makes consumption of fingerfoods impossible for ≥2 days. From all 16 patients living in the two group homes within the nursing home, 15 patients were included in the study after their family member signing informed consent.

### Intervention

Residents received all their meals from the nursing home including morning and afternoon snacks. In addition, during the intervention period fingerfoods were served daily around 4 pm as snacks, on top of their regular meals and snacks. The fingerfoods were provided by the general kitchen of the nursing home.

Before the start of the study, a wide array of different types of fruit and vegetable rich fingerfoods were pretested in a tasting session among the residents, including fresh fruit and vegetables presented, such as cherry tomatoes and paprika sticks, and fruit and vegetable rich baked goods. The fresh fruit and vegetable fingerfoods were poorly recognized by the residents and they often refused to taste them. Also, the kitchen staff indicated an unacceptable amount of preparation time related to fresh fruit and vegetables snacks. Another consideration to not continue with the fresh fruit and vegetables were the chewing and/or swallowing difficulties most residents had. The baked goods, however, were well accepted during the pretest despite their larger than normal amount of fruit and vegetables added. For the caregivers it was easy to offer the baked goods as it fit into the daily routine of the residents as they were used to receiving cake slices with their coffee and tea moments. Lastly, the baked goods fit with the residents’ increased preference for sweet. Therefore, we further developed and tested nutritious and fruit and vegetables rich baked goods in this study.

The researchers, dietician, and cooks of the nursing home developed four types of cake and three types quiche recipes based on the pretest; one type of fingerfood for each day of the week. The kitchen made these cakes and quiches in large batches and delivered them frozen to the group homes which improved cost efficiency and was compliant with the food safety regulation. To account for the chewing and/or swallowing difficulties the fingerfoods were free of nuts, bits and grits and large chunks of vegetables. The development and production the cost of the fingerfoods was €1,00 to €1,50 per piece during the pilot phase. The kitchen staff estimated to bring these prices down when embedding the process into a routine, using more seasonal ingredients and making larger batches.

To calculate nutritional contents of the fingerfoods, recipes of the seven types of fingerfoods were imported in the dietary assessment software Compl-eat (Version 1.0, Wageningen University, Wageningen) which is developed for the Dutch diet. Compl-eat is suitable for calculating nutritional contents based on food recipes: weights and amounts of ingredients can be imported and specific food characteristics, for example shrinkage of vegetables due to the cooking process can be taken into account. According to the calculations in Compl-eat, fruit and vegetable contents of all fingerfoods were around 50% (Table 1).

**Table 1:** Nutritional contents of all fingerfoods varieties per portion^*a*^

	Broccoli quiche	Beetroot brownie	Spinach quiche	Pumpkin spice cake	Vegetable quiche	Banana cake	Clafoutis pear
Portion size (g)	43.5	55.3	57.8	55.4	42.2	23.0	69.2
F/V content^*b*^ (g)	28.1	28.8	43.7	25.9	21.6	14.0	31.8
F/V content^*b*^ (%)	65%	53%	76%	47%	70%	52%	46%
Energy (kcal)	51	139	51	100	42	41	95
Protein (g)	2.9	1.5	2.5	2.6	2.0	1.1	2.3
Fats (g)	3.9	7.4	3.8	1.6	3.2	0.7	1.3
Saturated fats (g)	2.4	2.7	2.2	0.4	1.9	0.2	0.5
Carbohydrates (g)	0.6	15.9	1.1	18.2	1.0	7.3	18.3
Sugars (g)	0.4	12.0	0.7	10.4	0.9	4.4	15.0
Fibres (g)	0.7	1.8	1.0	1.3	0.6	0.6	0.7

^*a*^ *One portion was 5 × 5 cm, fingerfoods were served daily in two portions of 5 × 5 cm*

^*b*^ *F/V content = fruit and vegetable content*

To ensure fidelity a clear serving protocol was provided as described in the intervention and showcased in plain view of all the caregivers. The serving protocol instructions were: the caregivers provide the fingerfoods daily in a piece of circa 5 × 5 cm for each resident, warmed in the oven and served on small plates. The warming in the oven was to give the sensory aspect of filling the group home with the smell of fresh baked cake or quiche. If the first piece was consumed without protest a second piece was to be offered. The caregivers were asked to encourage the consumption of the fingerfoods, but not emphasize the health aspects; the fingerfoods were promoted as ‘tasty snack’ instead as they resembled a slice of cake or quiche.

### Outcome variables

#### Dose received by the residents

***Consumption of the fingerfoods***The caregivers were asked to record consumption of fingerfoods during the whole intervention period (every day of the week for 6 weeks long) using a checklist. On this checklist, caregivers could indicate how much of the offered fingerfoods was consumed by the resident (0, 0.5, 1, 1.5, 2 or more than 2 pieces) and provide optional remarks. All types of fingerfoods were weighed during both the first and last week of the intervention using a calibrated scale. The average of these measurements was used to indicate portion sizes. Based on the recipes and weight, nutritional contents were calculated using the dietary assessment software Compl-eat (Version 1.0, Wageningen University, Wageningen; [30]).

#### Fruit and vegetable consumption and food intake during dinner

Three-day food diaries were completed by the researchers to measure nutritional intake between 2.00 and 8.00 pm before and at the end of the intervention period (t0 and t6), to assess fruit and vegetable consumption and compensation behaviour of total intake during dinner. The focus of this measurement was on the afternoon fruit snack, tea time biscuit and the main evening meal (served between 5.30 and 6.30 pm), since this was the only meal where possible compensation for the fingerfoods was expected. The residents were randomly divided over three independent weekdays, thus the food record was registered for 3 days at t0 and t6 per resident. From the food diaries, energy intake and total fruit and vegetable consumption in the afternoon and evening were calculated using the dietary assessment software Compl-eat (Version 1.0, Wageningen University, Wageningen; 30).

#### Fidelity and appreciation by the caregivers

During the intervention period a researcher was present 1 day of the week to observe introduction of the fingerfoods, collect forms and offer assistance. A feedback form was provided in week 6 of the intervention to caregivers who had been involved in the distribution of the fingerfoods. This feedback form consisted of three open questions (positive and negative experiences with serving the fingerfoods and remarks for improvement about the individual fingerfoods) and three multiple-choice questions: [1] How did you experience serving the fingerfoods (easy, same as a regular snack, difficult), [2] What kind of atmosphere was created by (the eating of) fingerfoods (restful, restless, unchanged) and [3] How much time did the serving of fingerfoods take (it took little time, similar time as a regular snack, it took more time than regular snacks but this was no problem, it took too much time).

#### Other measurements

Baseline information (sex, age, dementia type, use of psychotropic medication, and use of other medication) of the residents was derived from patient information files and recorded anonymously. Nutritional status was measured at baseline by means of the Short Nutritional Assessment Questionnaire for Residential Care (SNAQ^rc^) [29], body weight, and body mass index (BMI). Body weight in kilograms was measured using a wheelchair scale. Body height was derived from patient information dossiers. BMI was calculated as weight (kg) divided by squared height (m) [30].

### Statistical analysis

SPSS (version 22, IBM SPSS Statistics, NY, USA) was used to analyse all data and for all tests, statistical significance was set at *P* < 0.05. Background characteristics and outcome variables were analysed. Normally distributed continuous variables were presented as means and standard deviations, non-normally distributed variables were presented as medians and interquartile ranges (IQRs). Categorical variables were presented as numbers and percentages. The differences in consumption between the types of fingerfoods were assessed with paired t-tests. Besides, energy intake and fruit and vegetable consumption between 2.00 and 8.00 pm was compared at t0 and t6 with paired t-tests.

## Results

### Study population

The residents mainly comprised females (93%; Table 2) and the majority of them was diagnosed with Alzheimer’s disease (*N* = 12, 80%). Residents consumed at baseline on average 30 g of fruit (median: 30.0 (34.5), 67 g of vegetables (median 67.1 (37.1)) and 312 kcal during dinner (median: 311.8 ± 115.4 kcal) at baseline.

**Table 2:** Baseline characteristics of 15 Dutch institutionalized patients with dementia

Baseline characteristic	Mean (SD), median (IQR) or N (%)
Sex (female) [*N*(%)]	14 (93%)
Age (years)	84.6 (9.8)
Dementia type [*N*(%)]	
Alzheimer	12 (80%)
Vascular	1 (7%)
Alzheimer + vascular	1 (7%)
Other	1 (7%)
Use of psychotropic medication, number [*N*(%)]^*a*^	
0	8 (53%)
1	4 (27%)
≥ 2	3 (20%)
Use of other medication, number [*N*(%)]^*a*^	
0–1	7 (47%)
2–4	7 (47%)
≥ 5	1 (7%)
Bodyweight (kg)	67.1 (16.8)
BMI (kg/m^2^)	24.5 ± 5.0
SNAQ^rc^ score [*N*(%)]	
Well nourished	3 (20%)
Moderately malnourished	6 (40%)
Severely malnourished	6 (40%)
Help needed with eating [*N*(%)]	
Yes	6 (40%)
No	9 (60%)
Energy intake during dinner (kcal)^*b,c*^	311.8 ± 115.4
Daily fruit consumption (g)^*c*^	30.0 (34.5)
Daily vegetable consumption (g)^*c*^	67.1 (37.1)

^*a*^ *Number of types of medication used*

^*b*^ *Dinner served daily between 5.30 and 6.30 pm*

^*c*^ *Average of 3-day food record between 2 and 8 pm*

### Dose received by the residents

#### Fingerfood consumption

Residents were offered two pieces of fingerfoods daily, of which on average 1.4 pieces (70.1 g) were consumed. These consumed portions contained on average 41 g of fruit/vegetables (Table 3). There were differences in serving weight between the types of fingerfoods and the portion sizes of the majority of these types increased during the intervention period due to changes in the kitchen. Dutch vegetable quiche was the fingerfood with the highest consumption (76% of the served portion consumed) and spinach quiche had the lowest consumption (64% consumed; Table 3).

**Table 3:** Average consumption per type of fingerfood and overall consumption

	Broccoli quiche	Beetroot brownie	Spinach quiche	Pumpkin spice cake	Vegetable quiche	Banana cake	Clafoutis pear	Overall
Portion size (pcs)^*a,b*^	1.3	1.4	1.3	1.5	1.5	1.4	1.5	1.4
Weight per portion consumed (g)^*a*^	58.0	77.9	73.9	81.1	63.7	35.0	101.0	70.1
Percentage consumed (%)^*a,c*^	66.6	70.5	64.0	73.2	75.5	71.4	73.0	70.5
Fruit/vegetable content (g)^*a*^	37.7	41.3	56.1	38.1	44.6	18.2	46.5	41.0

^*a*^ *Consumed on average*

^*b*^ *Portion size served = 2 pieces (pcs) fingerfood per day. One piece of fingerfood was 5 × 5 cm*

^*c*^ *Percentage of total average portion size served (2 pcs; total weight differed between every type of fingerfood)*

#### Changes in fruit and vegetable consumption and total food intake at dinner

Fruit and vegetable consumption was significantly higher (both together and separate) during the study period, from 91.7 g (SD = 28.6) at baseline to 165.4 g (SD = 46.3) after 6 weeks (mean difference = 73.7 ± 50.9 g, *P* < 0.001). The consumption of fingerfoods did not lead to significant caloric compensation during the main meals (mean difference = − 49.3 ± 114.8 kcal; *P* = 0.118).

### Fidelity and appreciation by the caregivers

During the 6 week period, fidelity of the intervention i.e. the distribution of the fingerfoods by the kitchen staff and caregivers, seemed high based on consumption rates as well as observations. The serving protocol seemed well accepted by the caregivers as this was similar to the way regular snacks were offered and clear instructions were provided in plain view of the caregivers. However, the observations showed that some caregivers had the tendency to present the fingerfoods with a fork which was not in accordance with the serving protocol.

In total, 20 caregivers and other staff members who had been involved in the fingerfoods project completed the three open questions on the feedback form, from which 12 caregivers also completed the three multiple choice questions (Table 4). Seven of the twelve caregivers indicated to have positive experiences in the open questions. They cited that most residents enjoyed receiving the fingerfoods which made it pleasant to offer the fingerfoods to them. The others gave a mixed review about the eating of fingerfoods by residents, depending on the type of fingerfood or the state of a resident. Most common complaint was that some types of fingerfoods became to soggy after heating, resulting in needing to be eaten with utensils, assistance or require extra cleaning. Within the multiple choice questions five out of twelve caregivers mentioned that the fingerfoods created a restless atmosphere (49%). Six caregivers indicated that the distribution of the fingerfoods was considered easy (50%) or similar (50%) to serving a regular snack. Regarding time, five caregivers (42%) indicated that it took little time or similar time as a regular snack, four caregivers (33%) said it took more time than regular snacks but this was no problem and three caregivers (25%) did indicate that it took too much time.

**Table 4:** Main results of multiple choice questions from the feedback form, filled in by caregivers and other personnel who had been involved distributing the fingerfoods to the residents (*N* = 12)

	N (%)
How did you experience serving the fingerfoods?	
Easy	6 (50%)
The same as serving a regular snack	6 (50%)
Difficult	0 (0%)
What kind of atmosphere was created by (the eating of) fingerfoods? ^*a*^	
Restfull	2 (18%)
The atmosphere was the same as before	4 (36%)
Restlessness	5 (46%)
How much time did the serving of fingerfoods take?	
It only took little time	3 (25%)
Similar time as a regular snack	2 (17%)
It took more time than regular snacks, but this was no problem	4 (33%)
It took too much time	3 (25%)

^*a*^ *Question was completed by 11 caregivers*

## Discussion

The results of this feasibility study showed that fingerfoods provided a positive means to enhance fruit and vegetable consumption and total food intake for institutionalized patients with dementia. The fingerfoods were well eaten by the residents and they did not compensate food intake during the rest of the day. Caregivers were generally positive and provided advice for improved implementation in the future.

In this feasibility study, providing fingerfoods as snacks led to an increased total food intake: patients were willing to consume the fingerfoods and did not compensate for the fingerfoods during dinner. This is in line with other studies looking at the effects of fingerfoods interventions in patients with dementia [20, 24, 31]. Providing fingerfoods as snacks may therefore be beneficial to increase food intake, especially when residents are not able to use cutlery but can still eat with their hands. Moreover, the residents in our study consumed on average 41 g of fruit or vegetables on top of their regular diet during the intervention period, which resulted in an increase of 45% of their total daily fruit and vegetable consumption. Fingerfoods may, therefore, be a helpful tool to counteract the negative consequences of a too low fruit and vegetable consumption in patients with dementia as well [13, 14]. However, it should be noted that the increase in fruit consumption is an overestimation, as one group home did not eat any fruit during the baseline measurements but did eat fruit daily in the measurement week at the end of the intervention. A reason for this difference in reported fruit consumption between the measurement weeks may be due to coincidental differences in the daily menu. Hence, to investigate the effects of fingerfoods on fruit and vegetable consumption, consumption should be measured over a longer period of time.

Fidelity by caregivers in serving the fingerfoods in this study seemed high. At the start of the study many of the caregivers were apprehensive to the concept of putting fruit and vegetables in baked goods, fearing that the combinations like chocolate beet brownies and banana bread would be too unfamiliar for the residents and therefore refused. However, residents were surprisingly accepting fingerfoods that looked like regular foodstuffs even if the ingredients were not standard, as long as the fingerfoods were presented as a nice snack. Also the tasting sessions with the caregivers before the start of the study helped to convince the caregivers to accept providing these fingerfoods to the residents. Most caregivers appreciated offering warm baked goods to the residents and the willingness of the residents to consume the fingerfoods supported this.

Results of this feasibility study showed that the caregivers were generally positive about proving the fingerfoods, depending on the type of fingerfood and state of a resident. For some caregivers, providing the fingerfoods as an extra snack moment on top of their daily routine was considered as a high burden on their time as it was slightly more labour intensive in preparing (heating in oven and serving on a plate) and cleaning up. Also, few residents were completely unable to eat by themselves from the start, which left the caregivers with an extra moment to handfeed them the fingerfoods. As fingerfoods are actually specifically developed for residents to be able to eat by themselves, future studies should pay attention to use fingerfoods that truly could be and are eaten by the residents themselves. If that is the case, providing fingerfoods should take no more time for the caregivers than usual snacks and thereby might be accepted more easily.

This feasibility study provided a few recommendations for future implementation of fingerfoods as snack in elderly homes. First, it is recommend that the caregivers are involved in the development of the fingerfoods through tasting sessions as their attitude during serving influences the acceptance of the residents. Also, collaboration between the caregivers and the kitchen team is suggested as it requires two teams to change their routine [32]. Second, the preparing and serving instructions should fit into the daily routines of the caregivers and should not be too strict, regarding for example serving time. Third, fingerfoods should be no more complex than eating a cookie for the residents and create no mess for the caregivers. With regard to future studies, the effectiveness of fingerfoods on the food intake, nutritional status and quality of life of patients in elderly homes should be further investigated. Using a randomized controlled design, the food intake and other outcomes of elderly receiving fingerfoods should be compared with a control group to estimate the effects of fingerfoods on this target group. Similar group homes for residents with dementia in the nursing home could be assigned as control group. Measurements of food intake and other outcomes should be measured over a longer period of time, while taking minimal effort from the caregivers.

### Strengths & limitations

To the best of our knowledge, this was the first feasibility study with fingerfoods rich in fruit and vegetables served as snacks in patients with dementia in the Netherlands. Another strength of this study was the focus on both the food intake by the residents and the effects on caregivers working in the nursing home. Since the study was implemented bottom-up, ideas and suggestions of people working in the field were taken into account before and during the study period, to improve the intervention and make the intervention more executable. The results and recommendations of this study can be used to improve the concept of fingerfoods as snacks, which can be helpful in implementing a successful larger scale fingerfoods intervention in further research.

A limitation of this study was the limited scale, caused by a small sample size and relatively short intervention period. Besides, slight changes were made in the daily eating rhythm and medication of patients during the study. These changes may have influenced the food intake of the residents and there was no control group in this study to account these slight changes. Another limitation is that the portion sizes of the fingerfoods slightly changed during the study period due to a changed procedure in the kitchen, which might have influenced the amount of consumed fingerfoods. For future studies on the effectiveness of fingerfoods on food intake, it therefore is recommended to provide fingerfoods on larger scale, over a longer period of time and to measure food intake over a longer period of time in both an intervention and control group to account for variations in the daily menu and intake of the elderly.

## Conclusion

The implementation of a fingerfoods intervention in a nursing home seems to be feasible for both patients with dementia and caregivers and could provide a pragmatic approach to enhance fruit and vegetable consumption and total food intake in institutionalized elderly. In further research, the recommendations derived from this feasibility study could be used for an effect evaluation and implementation of the fingerfoods concept on larger scale.

## Data Availability

The datasets generated and/or analysed during the current study are not publicly available due to them containing information that could compromise research participant privacy but are available from the corresponding author on reasonable request.

## Abbreviation

IQR: Interquartile range

## References

[1] WHO (2017). Dementia Fact sheet [Internet].

[2] Alexopoulos GS, Jeste DV, Chung H, Carpenter D, Ross R, Docherty JP. The expert consensus guideline series. Treatment of dementia and its behavioral disturbances. Introduction: methods, commentary, and summary. Postgrad Med. 2005;6:6–22. 17203561

[3] Prince M, Bryce R, Albanese E, Wimo A, Ribeiro W, Ferri CP (2013). The global prevalence of dementia: a systematic review and meta analysis. Alzheimers Dement [internet].

[4] Liu W, Cheon J, Thomas SA (2014). Interventions on mealtime difficulties in older adults with dementia: A systematic review. Int J Nurs Stud.

[5] Volicer L, Seltzer B, Rheaume Y, Karner J, Glennon M, Riley ME (1989). Eating difficulties in patients with probable dementia of the Alzheimer type. J Geriatr Psychiatry Neurol [Internet].

[6] Ikeda M, Brown J, Holland a J, Fukuhara R, Hodges JR (2002). Changes in appetite, food preference, and eating habits in frontotemporal dementia and Alzheimer’s disease. J Neurol Neurosurg Psychiatry.

[7] Papachristou I, Giatras N, Ussher M (2013). Impact of dementia progression on food-related processes: a qualitative study of caregivers’ perspectives. Am J Alzheimers Dis Other Dement.

[8] Pivi GAK, Bertolucci PHF, Schultz RR. Nutrition in severe dementia. Curr Gerontol Geriatr Res. 2012;2012. https://www.hindawi.com/journals/cggr/2012/983056/. 10.1155/2012/983056 PMC3356862 22645608

[9] Keller HH (2016). Improving food intake in persons living with dementia. Ann N Y Acad Sci.

[10] Jesus P, Desport JC, Massoulard A, Villemonteix C, Baptiste A, Gindre-Poulvelarie L (2012). Nutritional assessment and follow-up of residents with and without dementia in nursing homes in the Limousin region of France: a health network initiative. J Nutr Health Aging [Internet].

[11] Meijers JMM, Schols JMGA, RJG H (2014). Malnutrition in care home residents with dementia. J Nutr Health Aging [internet].

[12] Barratt J, Gatt J, Greatorex B, Scattergood J, Ryan C, Scordellis J. Using finger foods to promote independence, well-being and good nutrition in people with dementia. PSIGE Newsl. 2001;77:26–30.

[13] Raynaud-Simon A, Aussel C (2012). Fruit and vegetable intake in older hospitalized patients. Curr Opin Clin Nutr Metab Care [internet].

[14] Volkert D, Chourdakis M, Faxen-Irving G, Frühwald T, Landi F, Suominen MH (2015). ESPEN guidelines on nutrition in dementia. Clin Nutr.

[15] Ocke M, Buurma-Rethans E, De Boer E, Wilson-Van Den Hooven C, Etemad-Ghameshlou Z, Drijvers J. The diet of community-dwelling older adults. Results from the dutch national food consumption survey-2010-2012 [Internet]. Ann Nutr Metab. 2015;67:351–2 Available from: http://ovidsp.ovid.com/ovidweb.cgi? T=JS&PAGE=reference&D=emed14&NEWS=N&AN=72298874.

[16] Meeusen MJG, Bouwman E, Immink V, Sijtsema S. Kansen voor meer groenten en fruit in zorginstellingen. Wageningen; 2016.

[17] Zimmerman S, Williams CS, Reed PS, Boustani M, Preisser JS, Heck E (2005). Attitudes, stress, and satisfaction of staff who care for residents with dementia. Gerontologist..

[18] Chang CC, Roberts BL. Feeding difficulty in older adults with dementia. J Clin Nurs. 2008;17(17):2266–74. 10.1111/j.1365-2702.2007.02275.x 18705703

[19] Amella EJ, Grant AP, Mulloy C. Eating behavior in persons with moderate to late-stage dementia: assessment and interventions. J Am Psychiatr Nurses Assoc. 2008;13(6):360–7. 10.1177/1078390307309216 21672875

[20] Abdelhamid A, Bunn D, Copley M, Cowap V, Dickinson A, Gray L (2016). Effectiveness of interventions to directly support food and drink intake in people with dementia: systematic review and meta-analysis. BMC Geriatr [Internet].

[21] Jansen S, Ball L, Desbrow B, Morgan K, Moyle W, Hughes R. Nutrition and dementia care: informing dietetic practice. Nutr Diet. 2015;72(1):36–46.

[22] Crawley H, Hocking E. Eating well: supporting older people and older people with dementia. Caroline Walker Trust; 2011.

[23] VOICES (1998). Eating well for older people with dementia [Internet].

[24] Ford G (1996). Putting feeding back into the hands of patients. J Psychosoc Nurs Ment Health Serv.

[25] Jean LA (1997). “Finger food menu” restores independence in dining. Heal care food Nutr Focus.

[26] Nocent C, Martineau T. [Finger foods, old-age and autonomy] [French] Le manger-mains, vieillesse et autonomie. Soins Gerontol. 2011;(89):31–3. 21698964

[27] Linnan L, Steckler A (2002). Process evaluation for public health interventions and research: an overview. Process Evaluation for Public Health Interventions and Research.

[28] Durlak JA, DuPre EP. Implementation matters: a review of research on the influence of implementation on program outcomes and the factors affecting implementation. Am J Community Psychol. 2008;41(3-4):327. 10.1007/s10464-008-9165-0 18322790

[30] McWhirter JP (1994). Incidence and recognition of malnutrition in hospital. Clin Nutr.

[29] Kruizenga HM, de Vet HCW, van Marissing CME, Stassen EEPM, Strijk JE, Van Bokhorst-De Van Der Schueren MAE, et al. The SNAQRC, an easy traffic light system as a first step in the recognition of undernutrition in residential care. J Nutr Health Aging. 2009;14(2):83–89.10.1007/s12603-009-0147-120126953

[31] Heelan M, Prieto J, Roberts H, Gallant N, Barnes C, Green S. The use of finger foods in care settings: an integrative review. J Hum Nutr Diet. 2020;33(2):187–97. 10.1111/jhn.12725 31816144

[32] Dutch national health intstitutes (RIVM and Voedingscentrum). NEVO-tabel; Nederlands Voedingsstoffenbestand 2011. Den Haag; 2011.

